# Role of NKG2D, DNAM-1 and natural cytotoxicity receptors in cytotoxicity toward rhabdomyosarcoma cell lines mediated by resting and IL-15-activated human natural killer cells

**DOI:** 10.1007/s00262-015-1657-9

**Published:** 2015-02-18

**Authors:** Gerharda H. Boerman, Monique M. van Ostaijen-ten Dam, Kathelijne C. J. M. Kraal, Susy J. Santos, Lynne M. Ball, Arjan C. Lankester, Marco W. Schilham, R. Maarten Egeler, Maarten J. D. van Tol

**Affiliations:** 1 Section Immunology, Hematology, Bone Marrow Transplantation and Autoimmune Diseases, Department of Pediatrics, Leiden University Medical Center (LUMC), PO Box 9600, 2300 RC Leiden, The Netherlands; 2 Department of Pediatric Oncology, Academic Medical Centre (AMC), Amsterdam, The Netherlands; 3 Department of Stem Cell Transplantation and Haematology/Oncology, Hospital for Sick Children, University of Toronto, Toronto, Canada

**Keywords:** Rhabdomyosarcoma, Natural killer cells, Cytotoxicity, NK receptors, Immunotherapy

## Abstract

Children with advanced stages (relapsed/refractory and stage IV) of rhabdomyosarcoma (RMS) have a poor prognosis despite intensive chemotherapy and autologous stem cell rescue, with 5-year survival rates ranging from 5 to 35 %. Development of new, additional treatment modalities is necessary to improve the survival rate. In this preclinical study, we investigated the potential of resting and cytokine-activated natural killer (NK) cells to lyse RMS cell lines, as well as the pathways involved, to explore the eventual clinical application of (activated) NK cell immunotherapy. RMS cell lines (*n* = 3 derived from embryonal RMS and *n* = 2 derived from alveolar RMS) were susceptible to cytolysis mediated by resting NK cells, and this susceptibility was significantly increased using IL-15-activated NK cells. Flow cytometry and cytolytic assays were used to define the activating and inhibitory pathways of NK cells involved in recognizing and lysing RMS cells. NKG2D and DNAM-1 receptor-ligand interactions were essential in cytolysis by resting NK cells, as simultaneous blocking of both pathways resulted in almost complete abrogation of the cytotoxicity. In contrast, combined blocking of DNAM-1 and NKG2D only led to partial reduction of the lytic activity of IL-15-activated NK cells. In this respect, residual lysis was, at least partly, mediated by pathways involving the natural cytotoxicity receptors NKp30 and NKp46. These findings support further exploration of NK cell-based immunotherapy as adjuvant modality in current treatment strategies of RMS.

**Electronic supplementary material:**

The online version of this article (doi:10.1007/s00262-015-1657-9) contains supplementary material, which is available to authorized users.

## Introduction

Childhood rhabdomyosarcoma (RMS), a soft tissue malignant tumor of skeletal muscle origin, accounts for approximately 4 % of cancers among children aging 0–14 years with a peak incidence at 2–3 years [1]. Treatment and prognosis depend, in part, on the histology and molecular genetics of the RMS tumor. Patients diagnosed with alveolar RMS (ARMS) have a significantly worse prognosis than those with embryonal RMS (ERMS). Patients with primary metastatic disease or relapse have a survival rate of <35 % [2, 3]. Despite the development of more intensive treatment protocols, the survival rate has hardly improved in the past 20 years [1]. The poor prognosis in these patients makes it essential to pursue additional options for treatment, such as allogeneic hematopoietic stem cell transplantation (HSCT) and/or cell-mediated immunotherapy [4, 5].

The potential of immune cells to recognize and lyse tumor cells is currently explored to develop adjuvant immunotherapy against (pediatric) solid tumors. In this perspective, natural killer cells (NK cells) might be a promising tool. NK cells are bone marrow-derived lymphocytes defined by the expression of cell surface CD56 and the lack of CD3. NK cells do not bear clonally rearranged antigen-specific receptors and secrete immune-modulating cytokines upon activation. In addition, they display potent cytolytic activity against a wide range of tumor cells and virus-infected cells [6].

Reactivity of NK cells toward target cells is regulated by the balance between inhibitory and activating signals delivered through interactions of NK cell receptors and their respective ligands on target cells. NK cell inhibitory signals are provided by HLA class I molecules, which either bind to inhibitory Killer immunoglobulin-like receptors (KIRs) or NKG2A/CD94 on NK cells [7, 8]. Activating signals are delivered by stress-induced ligands, expressed by the tumor itself, such as the MHC class I chain-related gene A and B (MIC A/B) and UL16-binding proteins (ULBP1-6). The natural killer group 2 member D (NKG2D) receptor on the NK cell binds to these ligands, resulting in an activating signal [9, 10]. Other activating NK cell receptors include DNAX accessory molecule-1 (DNAM-1), which can recognize the CD112 (Nectin-2) and CD155 (PVR) molecules [11]. A third family of activating receptors consists of the natural cytotoxicity receptors (NCRs) NKp30, NKp44 and NKp46, of which the ligands are largely unknown [12].

To date, a small number of in vitro studies suggest that cytokine-activated (natural) killer cells can recognize and lyse RMS cell lines [13–15]. However, the pathways involved in the NK cell-mediated cytolytic activity toward RMS cells are largely unidentified. The aim of this study was to define the pivotal molecular interactions involved in recognition and lysis of RMS cells by resting and activated NK cells.

## Materials and methods

### Cell lines

The RMS cell lines A204, TE671 (ERMS) and the RH30, RH41 (ARMS) were all obtained from DSMZ (Braunschweig, Germany) and RD (ERMS) from ATCC (Manassa, VA, USA). Cells were maintained and cultured in DMEM Glutamax I high glucose (Life Technologies, Carlsbad, CA, USA), supplemented with 10 % heat-inactivated fetal calf serum (FCS, Greiner Bio-One, Kremsmuenster, Austria), 1 % MEM-non-essential amino acids and penicillin/streptomycin (100 U/mL and 100 μg/mL, respectively, both from Life Technologies). The NK-sensitive erythroleukemia cell line K562 and the NK-resistant Burkitt lymphoma cell line Daudi (both ATCC) were cultured in RPMI Glutamax I (Life Technologies) supplemented with 10 % heat-inactivated FCS and penicillin/streptomycin (100 U/mL and 100 μg/mL, respectively). Cell lines were routinely screened for mycoplasma contamination by PCR.

### Antibodies and flow cytometry

The following antibodies were used: CD16-FITC (clone #3G8), CD19-APC (#J4.119), CD56-RD1 (#N901), CD112-PE (R2.477.1), CD155 (#PV.404), Glycophorin A-PE (#KC16) and NKG2D-PE (#ON72) from Beckman-Coulter (Fullerton, California, USA); CD3-PerCPCy5.5 (#SK7), CD14/45 (lymphogate), CD33-PE (#P67.6), DNAM1-PE (#DX11), HLA-I-FITC (#G46-2.6) and MICA/B-PE (12D4) from BD Biosciences (San Jose, CA, USA); HLA-I (#W.6.32) from Biolegend (San Diego, CA, USA); MICA (#159227), ULBP-1 (#170818), ULBP-2 (#165903), ULBP-3 (#166510) from R&D Systems (Minneapolis, MN, USA). As isotype controls mouse IgG1/2a (R&D Systems) or mouse IgG2b (DAKO, Glostrup, Denmark) were used. Unlabeled antibodies were detected with goat anti-mouse Ig-APC (BD Pharmingen, San Jose, CA, USA). To study natural cytotoxicity receptors (NCRs) ligand expression, Fc fusion proteins constructed by connecting part of the NCR molecule to the Fc part of a human IgG1 molecule were applied (NKp30, NKp44 and NKp46 fusion proteins, 2.5 µg/mL; R&D Systems) and detected with goat antihuman Ig-A647 (Life Technologies). As isotype control, the anti-CD20 monoclonal antibody rituximab (2.5 µg/mL, Roche, Woerden, the Netherlands) was used. To demonstrate the specificity of the fusion proteins, we did perform an experiment in which staining of IL-15-activated NK cells for NKp30, NKp44 and NKp46 using MoAbs was specifically inhibited with the relevant fusion protein (25 µg/mL). The reduction of staining was 81 % (MFI) and 17 % (MFI) for NKp30-Fc and NKp46-Fc, respectively, and 94 % (% positive cells) for NKp44-Fc. Four-color flow cytometry was performed on a FACScalibur, and results were analyzed using Cellquest software (BD Biosciences).

### Isolation and culture of NK cells

Peripheral blood mononuclear cells (PBMCs) were obtained from healthy blood-bank donors by Ficoll density gradient separation. NK cells were isolated using the MACS NK Cell Isolation Kit according to the manufacturer’s protocol (Miltenyi Biotech, Bergisch Gladbach, Germany). NK cell purity was 94–99 %. Freshly isolated, resting NK cells were cultured for 2–5 weeks in AIM-V supplemented with glutamax I (Life Technologies), 10 % pooled human serum (Sanquin, Rotterdam, the Netherlands), penicillin/streptomycin (100 U/mL and 100 μg/mL, respectively) and 10 ng/mL recombinant human IL-15 (Cellgenix, Freiburg, Germany).

### Chromium release assays

Cytotoxicity was determined in a standard 4-h chromium release assay. Briefly, 1–3 × 10^6^ target cells were incubated with 100 μL sodium-51-Chromate (^51^Cr, 3.7 MBq, PerkinElmer, Waltham, MA, USA) for 1 h. Effector cells, resting or IL-15-activated NK cells, were incubated for 4 h with 2,500 target cells at eight effector:target (E:T) ratios. Maximum and spontaneous release was determined by incubating targets in hydrochloric acid (2 N HCl) or medium, respectively. Supernatants were harvested and measured in a gamma counter (Perkin Elmer). Percentage of specific lysis was determined as: (experimental counts-spontaneous release)/(maximum release-spontaneous release) × 100. In blocking experiments, before the addition of target cells, NK cells were incubated for 20 min with blocking antibodies specific for NKp30, NKp44 and NKp46 (all IgG1, clones #p30-15, #p44-8 and #9-E2, respectively, Biolegend); for DNAM-1 (IgG1; #DX11; BD Pharmingen); for NKG2D (IgG1; #149810; R&D) or for HLA class I (IgG1; #DX17; BD Pharmingen) at a concentration of 10 μg/mL.

### Co-cultivation

Freshly isolated NK cells were co-cultivated for 40 h with one of the RMS cell lines in the presence of IL-15 (10 ng/mL). NK cells (1 × 10^6^/well) were added to a semi-confluent layer of tumor cells, whereas controls consisted of NK cells cultured in the absence of RMS cell lines in medium only or in medium with IL-15. NK cells were harvested after 40 h and analyzed for their phenotype and cytolytic potential.

### Immunohistochemistry

Biopsies were taken at diagnosis from eight patients with ERMS. Four-μm sections containing representative tumor, as verified by an independent pathologist, were deparaffinized, and citrate antigen retrieval and endogenous peroxidases inactivation were performed. RMS cells in the biopsy sections were discerned by staining for myogenin (MYF4; #L026; Immunologic, Duiven, the Netherlands). Expression of the following ligands was assessed using rabbit polyclonal antibodies overnight at 4 °C: CD155 (hpa012568), ULBP-1 (hpa007547) and CD112 (hpa012759; all Sigma-Aldrich; Zwijndrecht, the Netherlands). MICA expression was assessed using a goat polyclonal antibody anti-MICA (AF1300, R&D Systems, Oxon, United Kingdom) overnight at room temperature on sections pre-treated with 10 % swine serum (Dako, Heverlee, Belgium) for 30 min at room temperature.

Antibody binding was detected by Liquid DAB + Substrate Chromogen System (Dako, Heverlee, Belgium) after applying Dako Envision + System–HRP-labeled Polymer Anti-Rabbit for the rabbit polyclonals or Dako LSAB + System–HRP for the goat polyclonal-treated sections. Testis was used as an internal positive control for the activating NK ligands. All samples were counterstained with hematoxylin, mounted with Pertex, and examined under a light microscope using CellB acquisition software (Olympus, Zoeterwoude, the Netherlands).

### Statistics

Statistical analyses were performed with GraphPad Prism version 6 using analysis of variance for comparing means between groups of samples or paired student *t* tests. Correction for multiple comparisons was applied using the Dunnett test correction. Error bars represent the standard error of the mean (SEM). A *p* value <0.05 was considered significant.

## Results

### RMS cell lines are highly susceptible to lysis by IL-15-activated NK cells

**Fig. 1:**
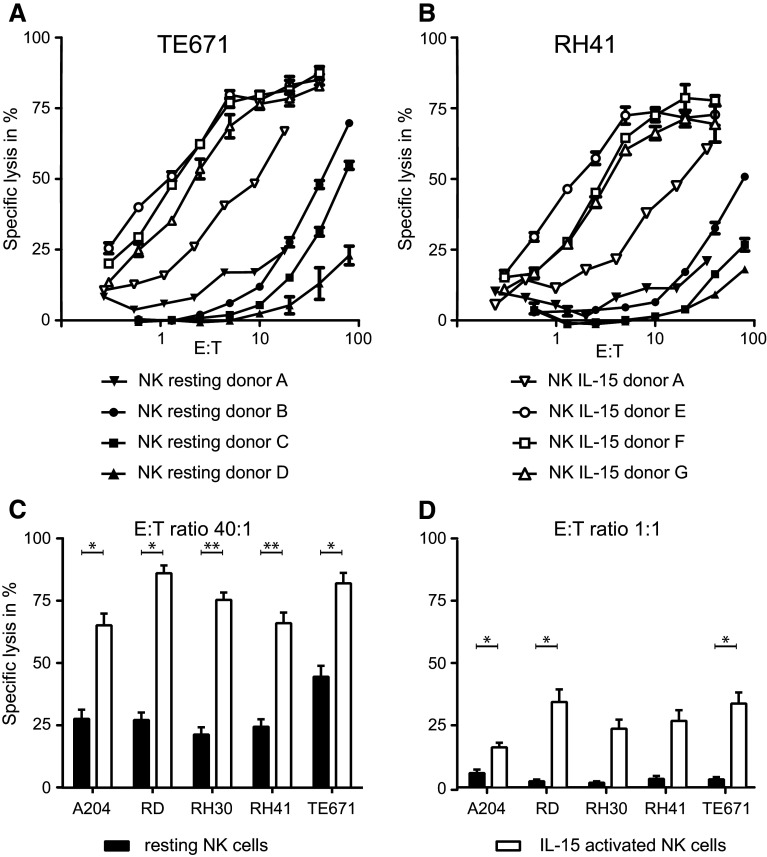
RMS cell lines are more susceptible to lysis by IL-15-activated than by resting NK cells. Specific lysis of rhabdomyosarcoma (RMS) cell lines TE671 (**a**) and RH41 (**b**) by purified, resting NK cells (*solid symbols*) or IL-15-activated NK cells (*open symbols*), measured in triplicate (SEM shown by *error bars*) in a 4-h ^51^Cr release assay. Data of the percentage specific lysis of RMS cells by resting (*solid bars*) and IL-15-activated NK cells (*open bars*) at effector:target ratio 40:1 (**c** resting NK cells: 16 donors; activated NK cells: 7 donors) and 1:1 (**d** resting NK cells: 16 donors; activated NK cells: 10 donors), respectively, are summarized. A *p* value <0.05 (indicated by *; <0.01 indicated by **) using paired *t* test was considered as a significant difference

We have investigated the in vitro lytic activity of NK cells from healthy donors (effectors) against RMS cell lines (targets) in a standard chromium release assay. NK cells were either used immediately after isolation (resting NK) or after activation with IL-15 for 2–5 weeks (IL-15-activated NK). Target cells were killed by resting NK cells (16 donors), although with a low efficacy as illustrated by the observation that high effector:target ratios (E:T > 40:1) were needed to obtain specific lysis above 25 % (Fig. 1a–c). Some variation in lytic activity of resting NK cells was observed among different donors (Fig. 1a, c).

In contrast, RMS susceptibility was strongly increased when using in vitro IL-15-activated NK cells (10 donors) as effectors. Il-15-activated NK cells efficiently recognized and lysed all RMS cell lines investigated, even at effector:target ratios as low as 1:1 (Fig. 1a, b, d). Moreover, the variation between donors, as observed for resting NK cells, was less evident after activation of NK cells by IL-15.

### Expression of NK cell receptor ligands on RMS cells

To explore the interaction pathways involved in the lysis of RMS cell lines by NK cells, expression patterns of activating and inhibitory ligands for NK cell receptors on RMS cell lines were investigated using flow cytometry (FACS). Both ERMS and ARMS cell lines heterogeneously expressed HLA class I, the NKG2A/CD94 and potential KIR ligand, and ligands for the various activating NK receptors (Table 1; Fig. 3a). In general, both DNAM-1 ligands (CD112 and CD155) were clearly expressed, whereas expression of NKG2D ligands, except for ULBP-3, was low or even absent on the majority of the RMS cell lines (Table 1). None of the RMS cell lines detectably expressed NKp30, NKp44 or NKp46 ligands using the Fc fusion proteins.

**Table 1:** Phenotypical characterization of RMS cell lines

	A204 (ERMS)	RD (ERMS)	RH30 (ARMS)	RH41 (ARMS)	TE671 (ERMS)
KIR ligand					
HLA-I	++	+	++	−	+
NKG2D ligands					
MICA/B	±	++	−	−	−
MICA	−	+	−	−	−
ULBP1	−	±	−	±	±
ULBP2	−	±	−	±	+
ULBP3	±	+	±	+	+
DNAM-1 ligands					
CD112	+	++	±	++	++
CD155	+	+	+	++	++
NCR ligands					
NKp30L	−	−	−	−	−
NKp44L	−	−	−	−	−
NKp46L	−	−	−	−	−

Expression of ligands for the inhibitory/activating NK cell receptors KIRs and the inhibitory receptor NKG2A/CD94 (HLA class I) and for the activating NK cell receptors (NKG2D, DNAM-1 and NCRs) was determined on RMS cell lines by flow cytometry. Mean fluorescence intensity (MFI) ratio of specific staining versus isotype control is depicted as:− = <2; ± = 2–5; + = 5–10; ++ = >10. *ERMS* embryonal rhabdomyosarcoma, *ARMS* alveolar rhabdomyosarcoma

**Fig. 2:**
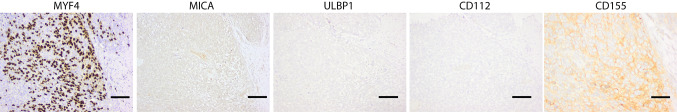
Immunohistochemistry of biopsy material for NKG2D and DNAM-1 ligands. Immunohistochemistry of NKG2D (MICA and ULBP1) and DNAM-1 (CD112 and CD155) ligands in biopsies of 8 ERMS patients was performed. A representative example (patient #5; Table 2) is shown for the ligands, which illustrate the heterogeneous staining patterns with correlations to the staining pattern of the tumor marker myogenin (MYF4)

To determine in vivo expression of the NKG2D and DNAM-1 ligands on RMS tumor cells, biopsy sections of 8 ERMS patients taken at diagnosis were stained for ULBP-1, MICA, CD112 and CD155 (Table 2; Fig. 2). Staining patterns of the different ligands were correlated with the expression pattern of the RMS tumor marker MYF4. One tumor section expressed only one ligand (MICA); in the other seven biopsies, expression of at least a NKG2D and a DNAM-1 ligand was observed.

**Table 2:** Expression of the NKG2D and DNAM-1 ligands on RMS tumor cells in biopsy sections

Patient #	NKG2D ligands		DNAM-1 ligands	
	MICA	ULBP1	CD112	CD155
1	++	+	−	+
2	+	−	+	−
3	+	+	+	−
4	+	−	−	−
5	+	−	−	++
6	++	−	+	−
7	−	+	−	+
8	+	−	+	−

Expression of ligands for the activating NK cell receptors (NKG2D, DNAM-1) was determined on RMS biopsy sections of 8 ERMS patients by immunohistochemistry (−: no staining or weak membrane staining in <10 % of the tumor cells; +: range of weak staining in at least 10 % of the tumor cells to intense membrane staining in ≤30 % of the tumor cells; ++: intense membrane staining in >30 % of the tumor cells)

### Lysis of RMS cell lines by resting NK cells is dependent on NKG2D and DNAM-1

**Fig. 3:**
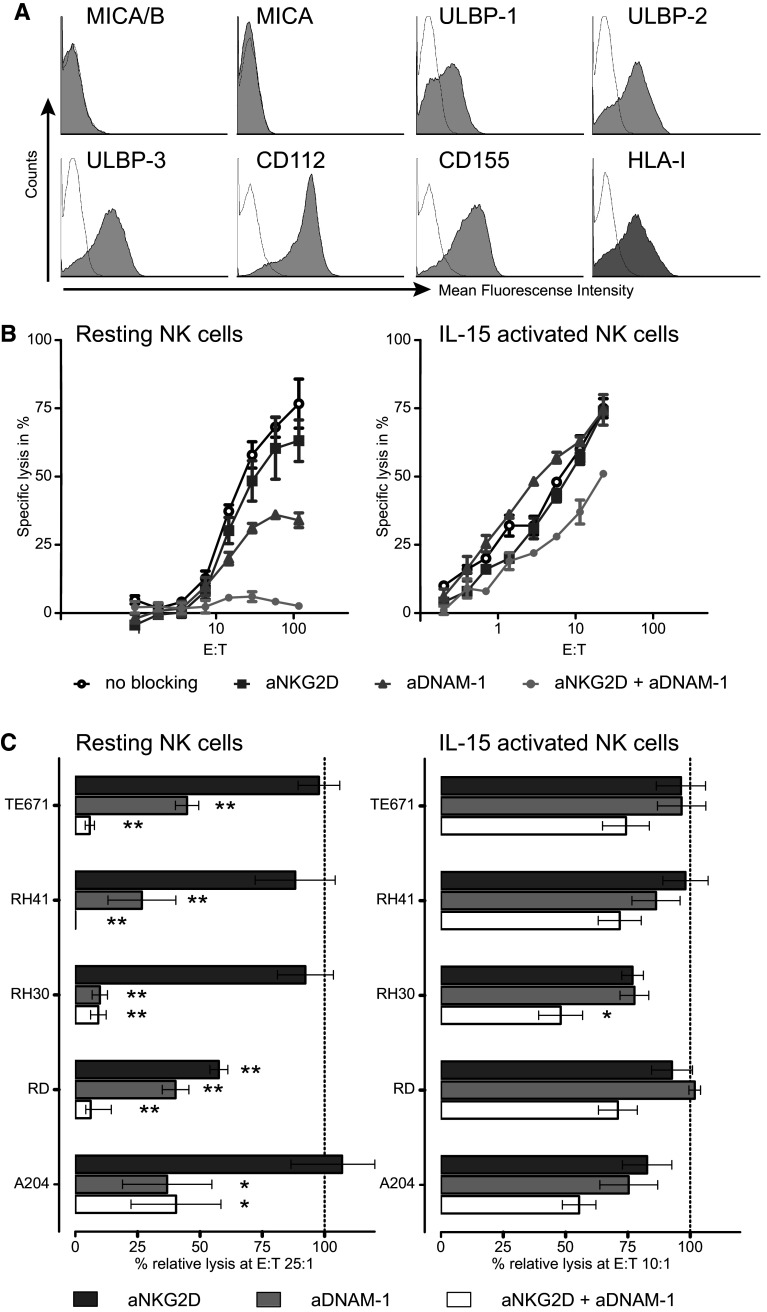
Lysis of RMS cell lines by resting NK cells is dependent on NKG2D and DNAM-1-mediated pathways. **a** Histograms of expression levels (*gray area* isotype control thin line) of NKG2D (MIC A/AB, ULBP1-3), DNAM-1 ligands (CD112 and CD155) and HLA-1 for the cell line TE671 measured by flow cytometry. **b** Representative specific lysis of the cell line TE671 by resting (*left panel*) and IL-15-activated NK cells (*right panel*) in the presence or absence (*open circle*) of blocking antibodies to NKG2D (*square*), DNAM-1 (*triangle*) or the combination of these two receptors (*closed circle*). *Error bars* represent the SEM of triplicates. **c** Combined data for the lysis of the RMS cell lines by resting (5 donors, E:T ratio 25:1, *left panel*) and IL-15-activated (6 donors, E:T ratio 10:1, *right panel*) NK cells in the presence of blocking antibodies to NKG2D (*dark gray bars*), DNAM-1 (*light gray bars*) and the combined antibodies (*white bars*). Data are depicted as percentage of the specific lysis obtained in the absence of blocking antibody. *Error bars* represent the SEM. Statistical analyses were performed using one-way ANOVA, followed by the Dunnett’s multiple comparisons test: *p* value <0.05 is indicated by *; <0.01 by **)

To investigate the contribution of the NKG2D and DNAM-1-mediated pathways to the interaction between NK cells and RMS cells, cytotoxicity assays were performed in the presence of blocking antibodies against the DNAM-1 and NKG2D receptor separately or in combination. In lysis assays performed with resting NK cells as effectors, blocking of DNAM-1 alone led to a more than 50 % reduction of the cytotoxicity toward all five cell lines, as depicted in Fig. 3b for the cell line TE671 and in Fig. 3c (left panel) at the E:T ratio 25:1 for all five RMS cell lines. In contrast, blocking of NKG2D alone had far less impact, with the exception of the cell line RD (Fig. 3c). This might be explained by the clear expression of the NKG2D ligand MICA/B, next to ULBP-3, particularly on this cell line (Table 1). However, there was no evident correlation between ligand expression and blocking pattern. The combination of DNAM-1 and NKG2D blocking led to an almost complete abrogation of killing by resting NK cells of all cell lines except for A204 (Fig. 3c, left panel).

### Lysis by IL-15-activated NK cells not only depends on DNAM-1 and NKG2D

**Fig. 4:**
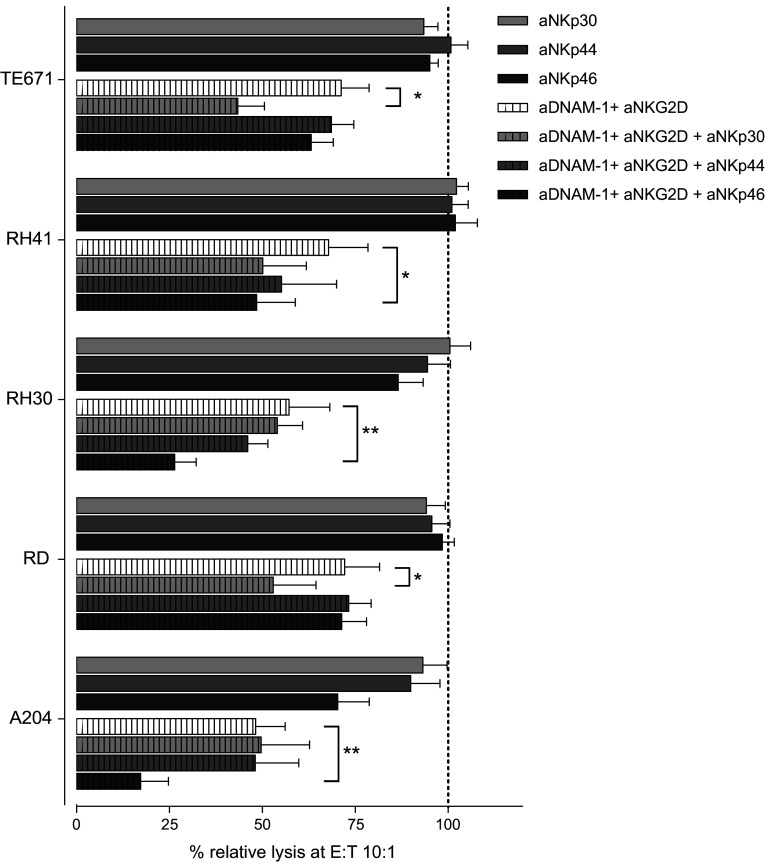
Effect of blocking of NCRs on lysis of RMS cell lines by IL-15-activated NK cells. Combined data for the lysis of the RMS cell lines by IL-15-activated NK cells (5–7 donors, E:T ratio 10:1) in the presence of blocking antibodies to NKp30, NKp44 and NKp46 alone (*solid bars*) or together with the combined blocking antibodies to DNAM-1 and NKG2D (*hatched bars*). Data are depicted as percentage of the specific lysis obtained in the absence of a blocking antibody. *Error bars* represent SEM. Statistical analyses comparing combined blocking of DNAM-1 and NKG2D in the presence of blocking of the indicated NCR with combined blocking of DNAM-1 and NKG2D alone were performed using one-way ANOVA, followed by the Dunnett’s multiple comparisons test: *p* value <0.05 is indicated by *; <0.01 by **)

In contrast to resting NK cells, lysis of RMS cell lines by IL-15-activated NK cells was only partially inhibited by combined blocking of NKG2D and DNAM-1 blocking (Fig. 3b, c right panels). This observation suggests that more pathways are involved in the interaction between IL-15-activated NK cells and RMS cells. In this respect, flow cytometry analyses indicated that the intensity of expression of not only DNAM-1 and NKG2D receptors but also of the NCRs, NKp30, NKp44 and NKp46 was increased upon IL-15 activation of NK cells (supplemental Table 1). To investigate the functional impact of the enhanced expression of the NCRs, blocking antibodies specific for the NCRs were added in the cytotoxicity assays. Despite undetectable expression of each of the NCR ligands on the RMS cell lines by FACS analysis (Table 1), a significant further reduction of cytolysis of three cell lines was seen when NKp46 was blocked on activated NK cells in combination with DNAM-1 and NKG2D blocking as compared to combined blocking of DNAM-1 and NKG2D alone (Fig. 4). Also, simultaneous blocking of NKp30 in combination with blocking of DNAM-1 and NKG2D led to a significant decrease of lysis in the cell lines TE671 and RD.

### Minor impact of HLA class I expression on NK cell-mediated cytolysis of RMS cell lines

Some HLA class I alleles are ligands of inhibitory and activating KIRs and the inhibitory NKG2A/CD94 receptor of NK cells. FACS analysis showed variable surface expression of HLA class I on the RMS cell lines, ranging from absent to strongly positive (Table 1).

**Fig. 5:**
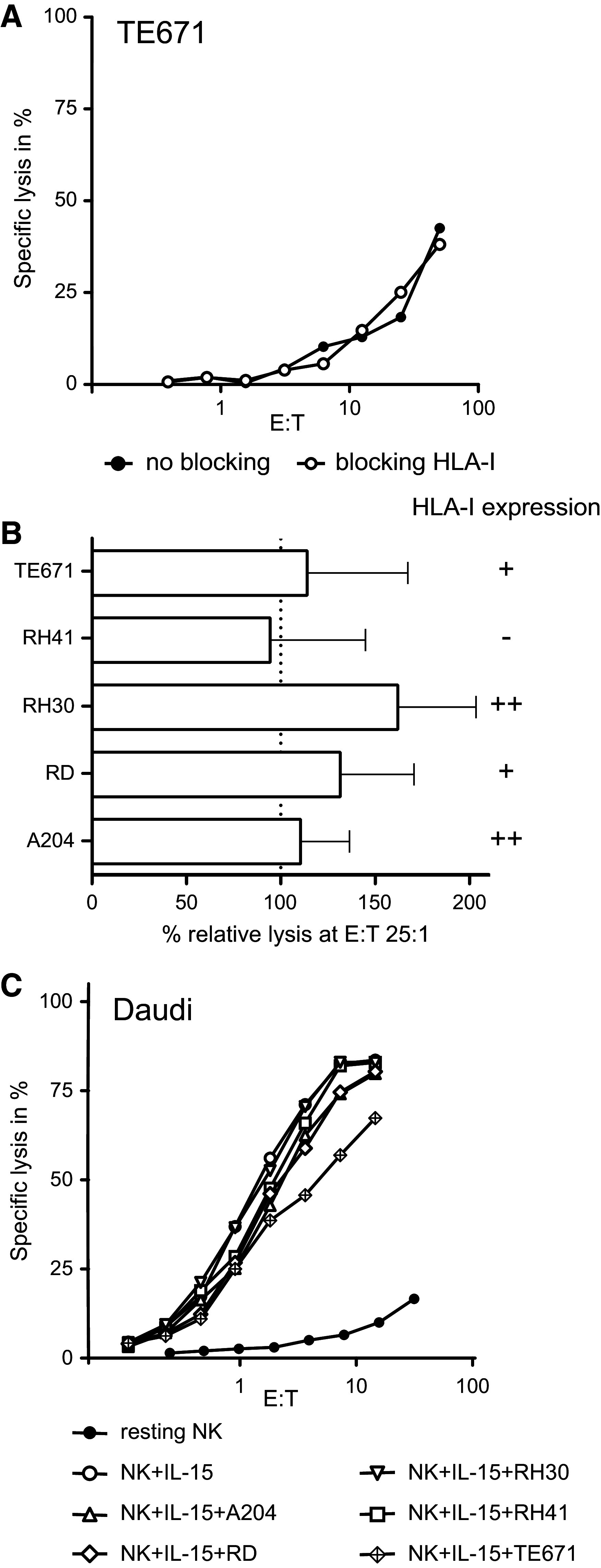
Neither HLA class I blocking nor co-cultivation with RMS cells affect NK cell-mediated cytotoxicity. **a** Specific lysis by resting NK cells of the cell line TE671 at different effector:target (E:T) ratios in the absence (*solid symbol*) or presence (*open symbol*) of anti-HLA class I blocking antibody Dx17. **b** Combined data of 3 independent assays and donors for the lysis of the RMS cell lines by resting NK cells at an E:T ratio of 25:1. Data are depicted as percentage of the specific lysis obtained in the absence of the anti-HLA class I antibody Dx17. *Error bars* represent SEM. **c** Purified NK cells were co-cultivated for 40 h with RMS cells in the presence of IL-15. Control cultures consisted of NK cells alone in the absence (*closed circles*) or presence of IL-15 (*open circles*). After culture, the NK cells were harvested and specific lysis of Daudi cells was measured at various effector:target (E:T) ratios

To investigate whether this HLA class I expression has an impact on susceptibility to NK cell cytotoxicity, the HLA class I-mediated interaction was blocked in vitro by pre-incubating the tumor cells with a monoclonal antibody directed to HLA class I (clone DX17) prior to the chromium release assay. After blocking of HLA class I, the sensitivity of RMS cell lines to cytolysis by resting NK cells remained unchanged (Fig. 5a) or only showed a trend to be increased (Fig. 5b). Moreover, no clear effect was seen in assays with IL-15-activated NK cells (data not shown). These results suggest that expression of HLA class I on RMS cells has no major impact on the interaction between allogeneic NK cells and RMS cell lines.

### Cytotoxicity of NK cells is not affected after co-cultivation with RMS cell in vitro

It cannot be excluded that RMS cells induce reduction of NK cell-mediated lysis, either via cell–cell contact or by production of soluble factors, representing a potential mechanism of RMS cells to escape from NK cell-mediated lysis. Hence, we performed assays to investigate possible changes in NK cell receptor expression and potential alteration of the lytic capacity of activated NK cells after co-cultivation with RMS cell lines [16]. After 40 h of co-cultivation with RMS cell lines, only a diminished expression of DNAM-1 receptor was observed on NK cells isolated from 1 out of the 4 donors investigated, irrespective of the RMS cell line used (Supplemental Figurer 1A). Most important, none of the co-cultivation conditions resulted in a decrease in the lytic potential of IL-15-activated NK cells toward K562 (target sensitive to resting and activated NK cells; data not shown) or Daudi (target sensitive to activated NK cells only; Fig. 5c and Supplemental Figure 1B).

## Discussion

In line with previous reports from our group investigating the in vitro cytotoxicity of NK cells against Ewing sarcoma and osteosarcoma [17, 18], we have demonstrated that cytokine-activated NK cells are much more effective in killing RMS target cells than resting NK cells. In general, the crucial role of DNAM-1 and NKG2D receptor-ligand interactions in NK cell-mediated killing of tumor cells is also observed in earlier reports on pediatric solid tumors and others tumors such as ovarian carcinoma, melanoma and acute myeloid leukemia [13, 17–23]. From these reports, the picture arises that the relative contribution of DNAM-1 and NKG2D receptor-ligand interactions varies between tumors and is different for resting and cytokine-activated NK cells. Similar to freshly isolated ovarian carcinoma [21], the DNAM-1 pathway seems relatively more important for the recognition of RMS cells by resting NK cells than the NKG2D-mediated pathway, illustrated by the observation that blocking of the DNAM-1 receptor resulted in significantly decreased lysis. Nevertheless, despite mostly weak or even undetectable expression of NKG2D ligands on RMS cells, simultaneous blocking of DNAM-1 and NKG2D led to almost complete abrogation of the cytotoxicity of resting NK cells. Of note, the overall intensity of expression of either DNAM-1 ligands or NKG2D ligands on the different RMS cell lines (Table 1) did not clearly correlate with the level of reduction of cytolysis by resting NK cells obtained after blocking with anti-DNAM-1 and anti-NKG2D, respectively (Fig. 3c).

The observation that blocking of both receptors on IL-15-activated NK cells only reduced cytotoxicity by 25–50 % indicates that more receptor-ligand pathways are involved in the cytolysis of RMS cells in this setting. In view of the observation that the expression intensity of NCRs was found to be increased on NK cells after IL-15 activation, and a role for NKp30 and NKp46 has been reported in killing of neuroblastoma cell lines [19], each of the NCRs, i.e., NKp30, NKp44 and NKp46, alone or in combination with DNAM-1 and NKG2D receptors, was blocked during the cytotoxicity assays. Remarkably, blocking of NKp30 or NKp46 receptors combined with blocking of DNAM-1 and NKG2D receptors resulted in an additional reduction of the lytic activity of IL-15-activated NK cells against 2 and 3 RMS cell lines, respectively, although expression of NCR ligands was not detected by FACS analysis. It remains speculative whether the expression intensity of the NCR ligands is too low for detection by flow cytometry using fusion proteins, whereas expression was observed in other types of tumors such as neuroblastoma and osteosarcoma cell lines (data not shown).

Not only activating receptors are important for the regulation and triggering of NK cells. Expression of HLA class I molecules is reported to mediate NK cell tolerance [12]. Despite heterogeneous expression levels of HLA class I, all RMS cell lines were sensitive to NK cell-mediated killing and no apparent correlation was observed between susceptibility to NK lysis and the expression level of HLA class I molecules. Furthermore, blocking of HLA class I only showed a trend to increase the susceptibility to lysis by NK cells. This lack of a significant effect could be explained by the likelihood that the signal of activating ligands is too strong to be surpassed by inhibitory ligands or the level of class I expression was too low to give sufficient inhibitory signals [24].

There is evidence that tumors are capable of escaping immunosurveillance by down regulating T cell or NK cell function [25, 26]. Raffaghello et al. [26] reported an immune evasion strategy employed by neuroblastoma cells through the production of a soluble form of MICA, which resulted in decreased expression of NKG2D receptors on CD8^+^ T cells of healthy controls and decreased cytotoxicity of activated NK cells. To investigate whether RMS cell lines are capable to inhibit NK cell function as an evasion strategy, RMS cells and NK cells were co-cultivated in the presence of IL-15. The results showed that RMS cells, in contrast to some osteosarcoma and neuroblastoma cell lines, at least in this in vitro setting, do not negatively affect cytolytic NK cell function [16, 27]. Neither the expression pattern of activating NK cell receptors nor the lytic capacity was altered after 40 h of co-cultivation. As we did not observed alteration in lytic capacity of NK cells after co-cultivation with RMS cells, the release of NKG2D ligands by RMS cell lines representing a possible immune escape mechanism was not investigated.

Since RMS cell lines are susceptible to (allogeneic) NK cell-mediated lysis, this tumor may be a suitable target for immunotherapy by adoptive transfer of (in vitro activated) NK cells. In haploidentical HSCT for children with hematologic malignancies and/or solid tumors, beneficial anti-tumor effects could be observed in the presence of an inhibitory KIR ligand mismatch between donor and recipient [28–31]. To further enhance this graft-versus-tumor effect, several studies were performed with transfer of in vitro activated NK cells. The strategies of adoptive NK cell therapy were found to be feasible and tolerable without enhancing the risk of graft-versus-host disease or other adverse events, and anti-tumor efficacy has been reported in some studies [32–35].

Taken together, a new treatment strategy using additional NK cell-based immunotherapy could provide a novel therapeutic option for children with relapsed or stage IV RMS. The in vitro data presented in this report motivate to further investigate whether RMS might be susceptible to NK cell-mediated killing in vivo as well.

### Electronic supplementary material

Below is the link to the electronic supplementary material.

Supplementary material 1 (PDF 134 kb)

## Abbreviations

ARMS: Alveolar rhabdomyosarcoma
DNAM-1: DNAX accessory molecule-1
E:T: Effector:target
ERMS: Embryonal rhabdomyosarcoma
FACS: Flow cytometry
FCS: Fetal bovine serum
HLA: Human leukocyte antigen
HSCT: Hematopoietic stem cell transplantation
IL-15: Interleukin 15
KIRs: Killer immunoglobulin-like receptors
MIC: MHC class I chain-related
MFI: Mean fluorescence intensity
NCRs: Natural cytotoxicity receptors
NK cells: Natural killer cells
NKG2D: Natural killer group 2 member D
PBMCs: Peripheral blood mononuclear cells
RMS: Rhabdomyosarcoma
ULBPs: UL16-binding proteins
