# Supplementary material for: Role of NKG2D, DNAM-1 and natural cytotoxicity receptors in cytotoxicity toward rhabdomyosarcoma cell lines mediated by resting and IL-15-activated human natural killer cells
Source: Cancer Immunol Immunother. 2015 Feb 18;64(5):573–83. doi: 10.1007/s00262-015-1657-9 (PMC4412555; doi:10.1007/s00262-015-1657-9)
Supplement: Supplementary file 1 — Supplementary material 1 (PDF 134 kb) [file 262_2015_1657_MOESM1_ESM.pdf]

Supplemental Table 1

Receptor	Resting NK	IL-15 activated NK	Fold increase
DNAM-1	24.9	37.6	1.5
NKG2D	30.3	157.4	5.2
NKp30	6.8	19.9	2.9
NKp44	13.1	93.3	7.1
NKp46	5.0	13.1	2.6

Supplemental Table 1: Up regulation of activating NK cell receptors after stimulation of NK cells with IL-15. Expression of the activating cell NK receptors (NKG2D, DNAM-1 and NCRs) was determined on freshly isolated NK cells (resting NK) and on these NK cells after activation with IL-15 (10 ng/mL) by flow cytometry (n=3). Mean fluorescence intensity (MFI) ratio of specific staining versus isotype control is shown.

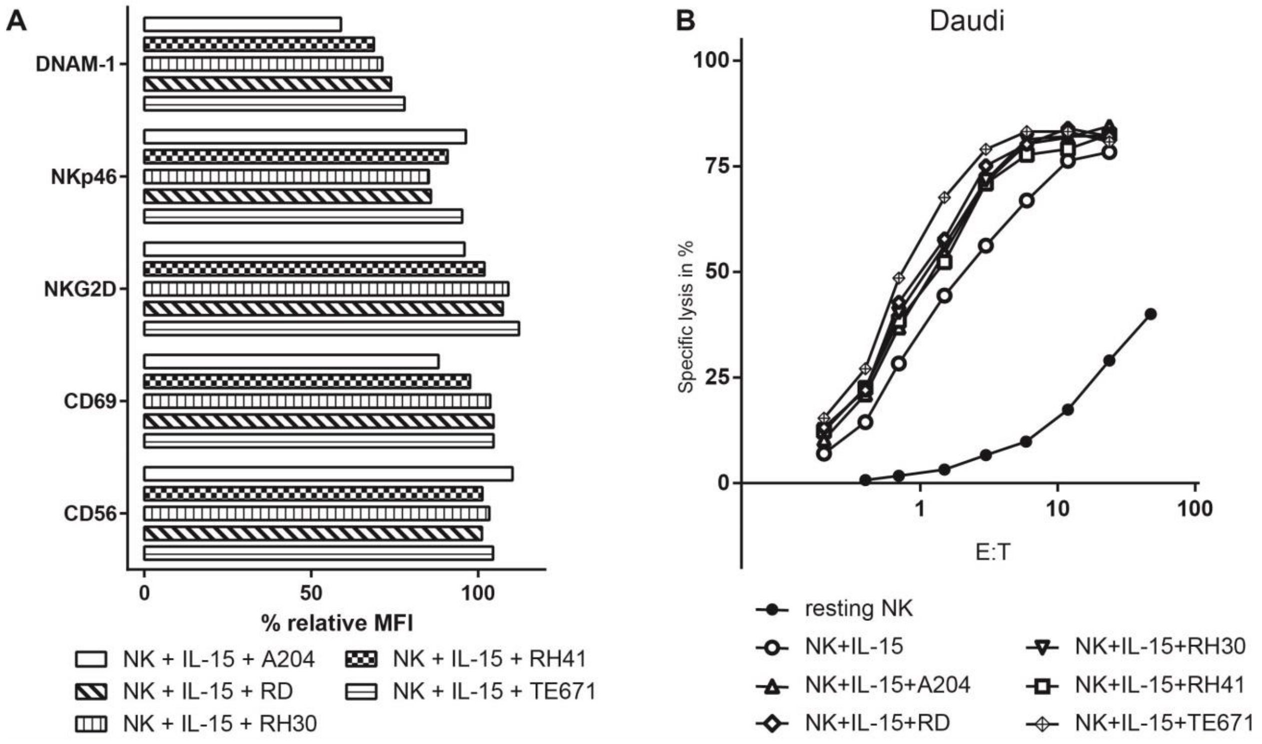

Supplemental Figure 1 Effect of co-cultivation of IL-15 activated NK cells with RMS cells on NK cell receptor expression and cytotoxicity.

Purified NK cells were co-cultivated for 40 hours with RMS cells in the presence of IL-15. After culture the NK cells were harvested and NK cell receptor expression (**A)** and specific lysis of Daudi cells (**B**) was measured.

(A) Data are depicted as percentage of the mean fluorescence intensity obtained for the control (NK cells cultured only presence of IL-15).

(B) Specific lysis was measured at various effector : target (E:T) ratios. Controls consisted of NK cells cultured alone in the absence (closed circles) or presence of IL-15 (open circles).
